# Supplementary material for: Effectiveness and Safety of Ayurvedic Medicines in Type 2 Diabetes Mellitus Management: A Systematic Review and Meta-Analysis
Source: Front Pharmacol. 2022 Jun 8;13:821810. doi: 10.3389/fphar.2022.821810 (PMC9213670; doi:10.3389/fphar.2022.821810)
Supplement: Supplementary file 1 [file DataSheet1.zip › Supplementary Material/Forest and Funnel Plots/Punica granatum/Insulin resistance.pdf]

Punica granatum - Insulin resistance

Study	Intervention N	Intervention Mean	Intervention SD	Comparator N	Comparator Mean	Comparator SD	Mean Diff. with 95% CI	Weight (%)
Babaeian 2013	23	3.75	1.85	22	5.02	3.14	-1.27 [-2.77, 0.23]	18.34
Sohrab 2014, Sohrab 2015	22	2.80	1.85	22	2.80	3.14	0.00 [-1.52, 1.52]	17.94
Faghihimani 2017	37	3.40	1.90	37	3.60	1.90	-0.20 [-1.07, 0.67]	32.31
Khajebishak 2019a, Khajebishak 2019b	26	4.67	2.67	26	4.42	3.75	0.25 [-1.52, 2.02]	14.59
Grabež 2020	19	6.70	1.96	18	5.14	2.93	1.56 [-0.04, 3.16]	16.82
**Overall**							0.00 [-0.79, 0.80]	

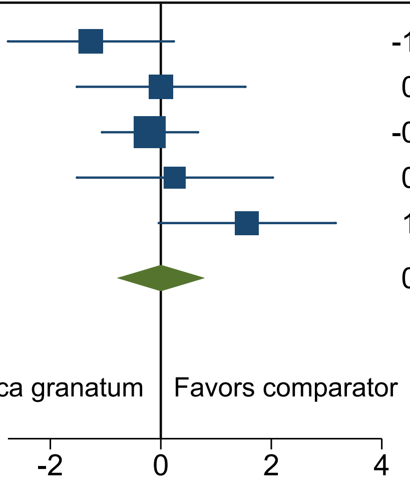

Heterogeneity: $\tau^2 = 0.32$, $I^2 = 38.81\%$, $H^2 = 1.63$

Test of $\theta_i = \theta_j$: Q(4) = 6.68, p = 0.15

Test of $\theta = 0$: z = 0.00, p = 1.00

Random-effects REML model
